# *Cis* interactions in the *Irf8* locus regulate stage-dependent enhancer activation

**DOI:** 10.1101/gad.350339.122

**Published:** 2023-04-01

**Authors:** Tian-Tian Liu, Feiya Ou, Julia A. Belk, Prachi Bagadia, David A. Anderson, Vivek Durai, Winnie Yao, Ansuman T. Satpathy, Theresa L. Murphy, Kenneth M. Murphy

**Affiliations:** 1 Department of Pathology and Immunology, Washington University in St. Louis School of Medicine, St. Louis, Missouri 63110, USA;; 2 Department of Computer Science, Stanford University, Stanford, California 94305, USA;; 3 Department of Pathology, Stanford University, Stanford, California 94305, USA

**Keywords:** enhancer cooperation, superenhancer, enhancer-associated lncRNA, IRF8, dendritic cell development

## Abstract

Individual elements within a superenhancer can act in a cooperative or temporal manner, but the underlying mechanisms remain obscure. We recently identified an *Irf8* superenhancer, within which different elements act at distinct stages of type 1 classical dendritic cell (cDC1) development. The +41-kb *Irf8* enhancer is required for pre-cDC1 specification, while the +32-kb *Irf8* enhancer acts to support subsequent cDC1 maturation. Here, we found that compound heterozygous Δ32/Δ41 mice, lacking the +32- and +41-kb enhancers on different chromosomes, show normal pre-cDC1 specification but, surprisingly, completely lack mature cDC1 development, suggesting *cis* dependence of the +32-kb enhancer on the +41-kb enhancer. Transcription of the +32-kb *Irf8* enhancer-associated long noncoding RNA (lncRNA) Gm39266 is also dependent on the +41-kb enhancer. However, cDC1 development in mice remained intact when Gm39266 transcripts were eliminated by CRISPR/Cas9-mediated deletion of lncRNA promoters and when transcription across the +32-kb enhancer was blocked by premature polyadenylation. We showed that chromatin accessibility and BATF3 binding at the +32-kb enhancer were dependent on a functional +41-kb enhancer located in *cis*. Thus, the +41-kb *Irf8* enhancer controls the subsequent activation of the +32-kb *Irf8* enhancer in a manner that is independent of associated lncRNA transcription.

## Abstract (precis)

In this study, Liu et al. describe how enhancers interact to temporally regulate *Irf8*, a transcription factor critical for dendritic cell lineage determination. Using a mouse model, they show that, in addition to its role in pre-cDC1 lineage commitment, the +41-kb *Irf8* enhancer also controls the activation of the +32-kb enhancer in *cis*, modifying chromatin accessibility and transcription factor binding required for cDC1 maturation.

Superenhancers were defined as large clusters of regulatory elements that drive expression of lineage-determining transcription factors (Hnisz et al. 2013). Individual constituent enhancers within a superenhancer can cooperate to activate target gene expression (Hnisz et al. 2017). Enhancer–enhancer interactions may be additive or synergistic and may occur concurrently or in a stage- or time-dependent manner (Blobel et al. 2021; Choi et al. 2021). In the *Myc* and α-globin superenhancers, individual constituents cooperated in an additive manner (Hay et al. 2016; Bahr et al. 2018). For myeloid-specific PU.1 regulation, the **−**12-kb and URE PU.1 enhancers cooperated synergistically (Leddin et al. 2011). Furthermore, a temporal enhancer hierarchy was suggested for the *Wap* superenhancer regulation, since mutation of the earliest constituent inactivated the entire superenhancer (Shin et al. 2016). All these studies had not tested whether enhancer–enhancer interactions occurred within a chromosome or, alternately, whether interchromosomal interactions were involved (Maass et al. 2019).

One previous study provided direct evidence for *cis*-dependent enhancer–enhancer interactions based on analysis of compound heterozygous enhancer mutations (Mehta et al. 2017). The *Gata2* locus is regulated by individual enhancer constituents, including the **−**77- and +9.5-kb enhancers (Grass et al. 2006). The +9.5-kb enhancer activates *Gata2* transcription in endothelium and hematopoietic stem cells (HSCs), whereas the **−**77-kb enhancer activates transcription in myeloid progenitors. *Cis*-dependent interactions were demonstrated by analysis of **−**77;+9.5 compound heterozygous mice, in which each chromosome harbors a different enhancer deletion (Mehta et al. 2017). This study found that the +9.5-kb *Gata2* enhancer alone is sufficient for HSC generation. In contrast, in order for the **−**77-kb enhancer to support myeloid lineage development, it must reside on the same chromosome as a functional +9.5-kb *Gata2* enhancer (Mehta et al. 2017).

Many different models have been proposed to explain enhancer function in gene control, in which the enhancer–promoter looping model enjoys the most experimental support (Panigrahi and O'Malley 2021). Enhancers may also regulate target gene expression via noncoding RNAs (ncRNAs) produced from the enhancer regions themselves (Statello et al. 2021). Active enhancers produce enhancer RNAs (eRNAs) that are generally bidirectionally transcribed, nonpolyadenylated, unspliced, and unstable. Enhancer regions are also enriched with lncRNA transcripts, which are mostly unidirectional, polyadenylated, and spliced (Ulitsky and Bartel 2013; Gil and Ulitsky 2018). The exact roles of the eRNAs or enhancer-associated lncRNAs in gene regulation remain unclear (Kim et al. 2010; Mowel et al. 2018). A central question is whether the ncRNA transcript itself or the transcription across enhancers is what directly activates the enhancer or, alternately, whether the ncRNA production is simply a reflection of the active chromatin state of the enhancer region (Arnold et al. 2020).

In some cases, the eRNA or enhancer-associated lncRNA transcripts are functional and can act both in *cis* and in *trans* to regulate gene expression. Several enhancer-associated ncRNA transcripts were identified that regulate their neighboring genes in *cis*. The mechanisms include regulating chromatin accessibility, chromatin architecture, and the recruitment of transcription machinery or cofactors. For example, the enhancer-associated lncRNA ^DRR^RNA was found to promote chromatin accessibility at the *Myog* locus (Mousavi et al. 2013). The inducible *Ifnb1* and *Tnfsf10* enhancer-associated ncRNA transcripts were shown to promote the physical interaction between enhancers and their target promoters (Kim et al. 2018). Enhancer-associated ncRNAs can increase RNA polymerase II (RNAPII) occupancy at protein-coding loci (Mousavi et al. 2013) or regulate RNAPII pause release by acting as a decoy for the negative elongation factor complex (Schaukowitch et al. 2014). Additionally, enhancer-associated ncRNAs can recruit transcription factors or several general cofactors, including YY1 (Sigova et al. 2015), cohesin (Li et al. 2013), Mediator (Lai et al. 2013), CBP/p300 (Bose et al. 2017), and BRD4 (Rahnamoun et al. 2018), to augment transcriptional activation through the regulation of enhancer–promoter looping, chromatin remodeling, and transcriptional elongation. Enhancer-associated lncRNA has also been found to act in *trans* to regulate genes on different chromosomes (Tsai et al. 2018). A *MyoD* enhancer-associated lncRNA mediates cohesin recruitment to the *Myogenin* gene locus in *trans* to control myogenic differentiation (Tsai et al. 2018).

In other cases, it is the process of transcription and splicing rather than the ncRNA transcript itself that functions to regulate the target gene expression. For example, transcription of the *Hand2* enhancer-associated lncRNA *Uph* establishes a permissive chromatin environment at the enhancer to promote *Hand2* expression during heart development (Anderson et al. 2016). Transcription of the *Bcl11b* enhancer-associated lncRNA *ThymoD* instructs chromatin folding and compartmentalization to regulate *Bcl11b* enhancer–promoter communication during T-cell development (Isoda et al. 2017). The splicing of the lncRNA *Blustr* was also found to play critical roles in activating the neighboring gene, *Sfmbt2* (Engreitz et al. 2016).

Finally, in some cases, it is only the DNA *cis* element residing within the region of an enhancer-associated ncRNA that is required for enhancer function. For example, the *Cdkn1b* enhancer-associated lncRNA *Lockd* transcripts can be truncated by insertion of polyadenylation cassettes without affecting *Cdkn1b* expression (Paralkar et al. 2016). *Cis* activation of *Bend4* was also found to be independent of mature lncRNA *Bendr* transcripts or significant *Bendr* transcription (Engreitz et al. 2016).

The *Irf8* locus is the top-ranked superenhancer in cDC1 (Grajales-Reyes et al. 2015), a dendritic cell lineage that supports in vivo priming of CD8 T cells against viruses and tumors (Anderson et al. 2021; Murphy and Murphy 2022). IRF8 is the lineage-determining transcription factor for cDC1 development (Aliberti et al. 2003; Tsujimura et al. 2003). Three constituent enhancers in the *Irf8* superenhancer have been identified that sequentially regulate *Irf8* expression at different stages of cDC1 development (Durai et al. 2019; Murakami et al. 2021). The +56-kb enhancer initiates *Irf8* expression in multipotent progenitor and is required for IRF8 expression in the monocyte/dendritic cell progenitor (MDP) (Murakami et al. 2021). The E-protein-dependent +41-kb *Irf8* enhancer becomes active and increases IRF8 levels during the transition from MDPs to common DC progenitors (CDPs) (Durai et al. 2019). In addition, the +41-kb enhancer is required for specification of the pre-cDC1 progenitor from within the CDP and remains active to support *Irf8* expression in plasmacytoid DCs (pDCs) (Durai et al. 2019). Finally, the BATF3-dependent +32-kb *Irf8* enhancer acts after pre-cDC1 specification to support *Irf8* autoactivation in the pre-cDC1 progenitor and mature cDC1 (Durai et al. 2019).

This study was prompted by our unexpected observation that compound heterozygous mutations in the +32- and +41-kb *Irf8* enhancers caused complete loss of cDC1 development despite the presence of one intact +32-kb enhancer. Here, we evaluated the potential role for the +32-kb *Irf8* enhancer-associated lncRNA Gm39266 and examined the basis for this enhancer–enhancer *cis* interaction.

## Results

### The +41-kb *Irf8* enhancer *cis*-regulates +32-kb *Irf8* enhancer activity

**Figure 1.**
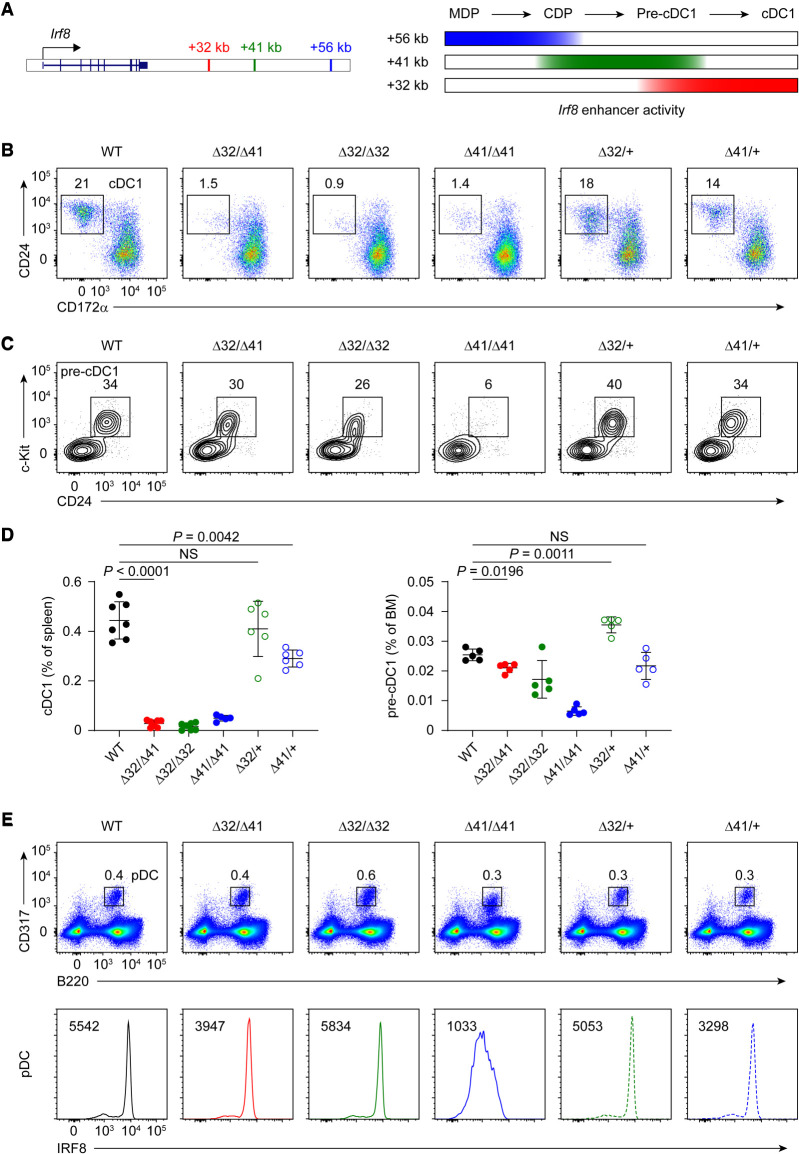
The +41-kb *Irf8* enhancer *cis*-regulates +32-kb *Irf8* enhancer activity. (*A*) Schematic of *Irf8* enhancer activity during cDC1 development. (*B*) Representative flow plots showing splenic cDC1s in the indicated *Irf8* enhancer mutant mice. (*C*) Representative flow plots showing pre-cDC1 progenitors in bone marrow (BM) of the indicated *Irf8* enhancer mutant mice. (*D*) Frequency of splenic cDC1s and BM pre-cDC1 progenitors in the indicated *Irf8* enhancer mutant mice. Data are pooled from six independent experiments for splenic cDC1s and from three independent experiments for BM pre-cDC1 progenitors. (*E*) Representative flow plots showing splenic pDCs (*top*) and intracellular staining for IRF8 in pDCs (*bottom*) from the indicated *Irf8* enhancer mutant mice. Data shown are one of six similar experiments for splenic pDCs and one of two similar experiments for IRF8 expression. Data are mean ± SD. (NS) Not significant. (*D*) Brown−Forsythe and Welch ANOVA with Dunnett's T3 multiple comparisons test.

During cDC1 development, *Irf8* expression is first supported by the E-protein-dependent +41-kb *Irf8* enhancer in the early DC progenitors and later requires the BATF3-dependent +32-kb *Irf8* enhancer in the specified pre-cDC1 progenitors and mature cDC1s (Fig. 1A; Durai et al. 2019). To explore the potential enhancer–enhancer interactions in regulating *Irf8* expression, we generated compound heterozygous mice bearing +32- and +41-kb enhancer deletions on different *Irf8* alleles (Δ32/Δ41 mice). As we previously reported, in mice with homozygous deletions of the +41-kb enhancer (Δ41/Δ41 mice), both the pre-cDC1s and mature cDC1s fail to develop (Fig. 1B–D). Also, mice with homozygous deletions of the +32-kb enhancer (Δ32/Δ32 mice) have normal pre-cDC1s but lack mature cDC1 development (Fig. 1B–D). However, in Δ32/Δ41 mice, we observed the persistent loss of mature cDC1 development (Fig. 1B,D) despite normal pre-cDC1 specification (Fig. 1C,D). Thus, the one copy of the +41-kb *Irf8* enhancer in Δ32/Δ41 mice is functional in supporting pre-cDC1 specification. Also, the single copy of the +41-kb *Irf8* enhancer in Δ32/Δ41 mice supports similar levels of IRF8 expression in pDCs as in Δ41/+ mice, while pDCs from both Δ32/Δ41 and Δ41/+ mice show slightly reduced IRF8 expression compared with WT mice (Fig. 1E). In contrast, the one copy of the +32-kb *Irf8* enhancer in Δ32/Δ41 mice fails to support cDC1 maturation, compared with normal cDC1 development in Δ32/+ mice (Fig. 1B,D). Also, the single copy of the +32-kb *Irf8* enhancer in Δ32/Δ41 mice cannot maintain high IRF8 expression level in pre-cDC1s as in Δ32/+ mice (Supplemental Fig. S1).

### The +41-kb *Irf8* enhancer is required for lncRNA Gm39266 expression

**Figure 2.**
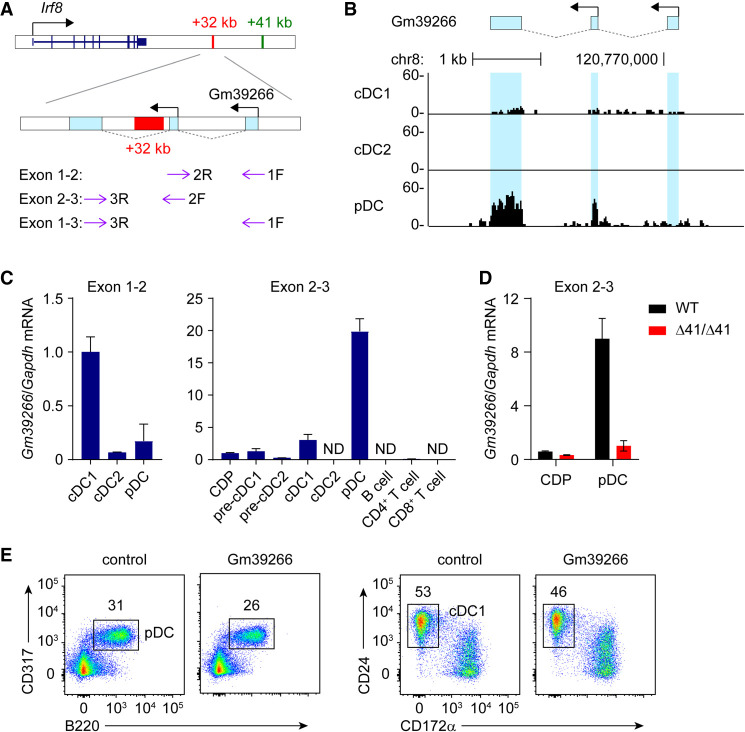
The +41-kb *Irf8* enhancer is required for lncRNA Gm39266 expression. (*A*) Schematic of the mouse *Irf8* locus. Blue boxes denote exons of lncRNA Gm39266. Arrows indicate oligonucleotides used for detecting Gm39266. (*B*) RNA-seq tracks display Gm39266 expression in cDC1, cDC2, and pDC. (*C*) Gm39266 transcripts, measured by RT-qPCR, in the indicated cell types isolated from BM (CDPs, pre-cDC1s, and pre-cDC2s) and spleens (all other cell types) of WT mice. Data shown are one of two similar experiments. (*D*) Gm39266 transcripts, measured by RT-qPCR, in CDPs and splenic pDCs isolated from WT and Δ41/Δ41 mice. Data shown are one of three similar experiments. (*E*) Representative flow plots showing pDCs and cDC1s differentiated from Flt3L cultures of CD117^hi^ BM progenitors retrovirally expressing Gm39266.

In exploring the mechanism of the *cis*-regulation between +41- and +32-kb *Irf8* enhancers, we identified a lncRNA, Gm39266, spanning the +32-kb *Irf8* enhancer region using the annotation of the mouse genome provided by GENCODE (Fig. 2A). The +32-kb *Irf8* enhancer is located within intron 2 of lncRNA Gm39266, which is a spliced, 744-nt transcript that could be amplified using oligo(dT) primers, indicating that it undergoes polyadenylation. Gm39266 comprises differentially expressed isoforms. RNA-seq analysis showed that a short isoform comprising exons 2 to 3 of Gm39266 was highly expressed in pDCs but not in cDC1s or cDC2s (Fig. 2B). To confirm this expression pattern, we designed oligonucleotide primers that selectively detect the full-length of Gm39266 (exon 1–2) or both full-length and short isoforms (exon 2–3) (Fig. 2A). Similar to the RNA-seq data, the oligonucleotide primer pair exon 2–3 detects high levels of Gm39266 transcripts in pDCs and only low levels of Gm39266 expression in CDPs, pre-cDC1 progenitors, and mature cDC1s (Fig. 2C). In contrast, the full-length Gm39266 isoform is selectively expressed in cDC1s but not in cDC2s or pDCs (Fig. 2C). These results suggest that pDCs highly express a short isoform of lncRNA Gm39266, while cDC1s express a less abundant but full-length Gm39266 isoform.

Interestingly, the expression of the Gm39266 short isoform shows the same pattern as the +41-kb *Irf8* enhancer activity. Both are highly active in pDCs but not in cDC1s or cDC2s. This observation prompted us to ask whether the +41-kb *Irf8* enhancer regulates Gm39266 expression. To test this idea, we asked whether Gm39266 transcription was maintained in mice lacking the +41-kb enhancer. RT-qPCR analysis showed that pDCs from Δ41/Δ41 mice show a substantial reduction in Gm39266 expression (Fig. 2D). This result indicates that the +41-kb *Irf8* enhancer regulates transcriptional activity at the +32-kb *Irf8* enhancer region.

### Transcription across the +32-kb *Irf8* enhancer is not required for its enhancer activity

Since the +41-kb *Irf8* enhancer is required for the transcription of lncRNA Gm39266, and since transcription of Gm39266 between exons 2 and 3 crosses right over the +32-kb enhancer, we wondered whether the +41-kb enhancer-dependent transcription or transcripts of Gm39266 are required for the activation of the +32-kb *Irf8* enhancer. To test this hypothesis, we first evaluated the effect of Gm39266 transcripts in cDC1 development. We found that retroviral expression of Gm39266 did not influence cDC1 or pDC development (Fig. 2E), suggesting that the Gm39266 transcript itself is not active in driving cDC1 development.

**Figure 3.**
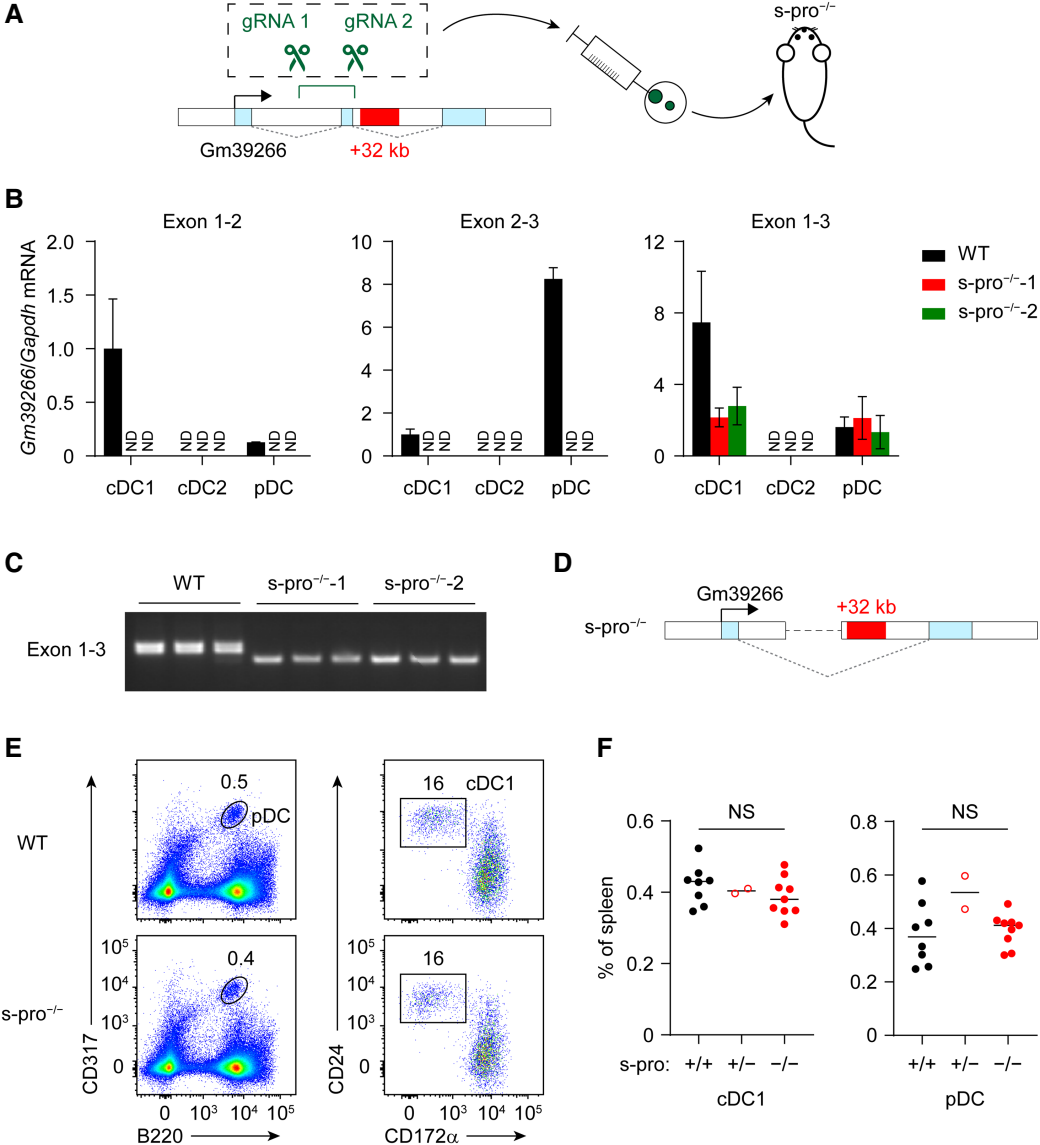
Deletion of Gm39266 exon 2 leads to alternative splicing and does not eliminate Gm39266 transcription across the +32-kb *Irf8* enhancer. (*A*) Targeting strategy of Gm39266 exon 2 short promoter deletion (s-pro^–/–^) mice. (*B*) Gm39266 transcripts, measured by RT-qPCR, in cDC1s, cDC2s, and pDCs isolated from WT and s-pro^–/–^ mice. Data shown are one of two similar experiments. (*C*) RT-PCR of Gm39266 in cDC1s isolated from WT and s-pro^–/–^ mice. Data shown are one of two similar experiments. (*D*) Schematic showing Gm39266 alternative splicing in s-pro^–/–^ mice. (*E*) Representative flow plots showing splenic pDCs and cDC1s in WT and s-pro^–/–^ mice. (*F*) Frequency of splenic cDC1s and pDCs in WT, s-pro^+/–^, and s-pro^–/–^ mice. Data are pooled from four independent experiments. Data in *B* are mean ± SD. Center values in *F* indicate the median. (NS) Not significant. (*F*) Brown−Forsythe and Welch ANOVA with Dunnett's T3 multiple comparisons test.

Next, to ask whether the lncRNA Gm39266 transcripts or transcription across the +32-kb *Irf8* enhancer region is required for +32-kb *Irf8* enhancer activity, we used CRISPR/Cas9 editing to delete exon 2 and the promoter of the short Gm39266 isoform in mice in order to eliminate both the full-length and the short Gm39266 isoforms in vivo (Fig. 3A; Supplemental Fig. S2A). We confirmed the complete deletion of the Gm39266 exon 2 genomic region in short promoter deletion (s-pro^–/–^) mice (Fig. 3B). However, we noted that splicing from exon 1 to exon 3 could still occur, since RT-PCR using oligonucleotide primers located in Gm39266 exons 1 and 3 was able to amplify a shorter DNA product in cDC1 from s-pro^–/–^ mice (Fig. 3B,C). In addition, sequencing of this shorter DNA product demonstrated the alternative splicing from exon 1 to exon 3 (Fig. 3D). In summary, deletion of exon 2 and the short isoform promoter does not completely eliminate Gm39266 transcription across the +32-kb *Irf8* enhancer region. Furthermore, +32-kb *Irf8* enhancer activity remains intact in s-pro^–/–^ mice, since we observed normal cDC1 development in these mice (Fig. 3E,F).

**Figure 4.**
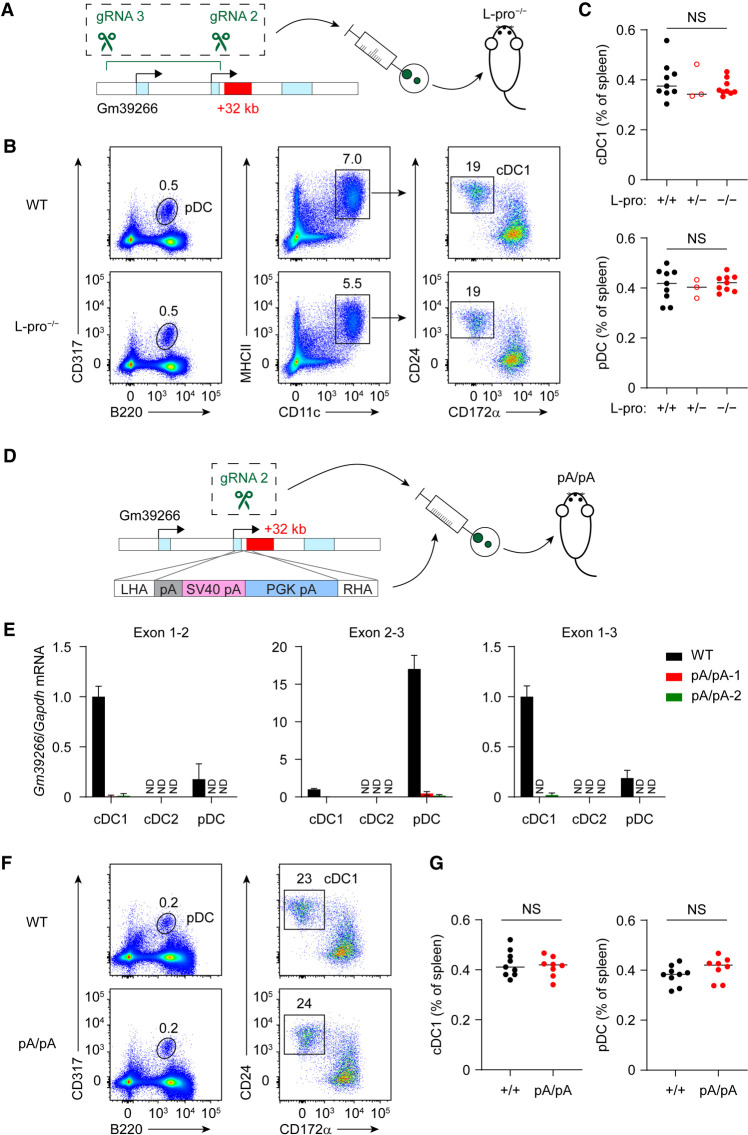
Transcription across the +32-kb *Irf8* enhancer is not required for its normal enhancer activity during cDC1 development. (*A*) Targeting strategy of Gm39266 exon 1–2 long promoter deletion (L-pro^–/–^) mice. (*B*) Representative flow plots showing splenic pDCs and cDC1s in WT and L-pro^–/–^ mice. (*C*) Frequency of splenic cDC1s and pDCs in WT, L-pro^+/–^, and L-pro^–/–^ mice. Data are pooled from three independent experiments. (*D*) Targeting strategy of Gm39266 3× polyA knock-in (pA/pA) mice. (*E*) Gm39266 transcripts, measured by RT-qPCR, in cDC1s, cDC2s, and pDCs isolated from WT and pA/pA mice. Data shown are one of two similar experiments. (*F*) Representative flow plots showing splenic pDCs and cDC1s in WT and pA/pA mice. (*G*) Frequency of splenic cDC1s and pDCs in WT and pA/pA mice. Data are pooled from three independent experiments. Center values in *C* and *G* indicate the median. Data in *E* are mean ± SD. (NS) Not significant. (*C*) Brown−Forsythe and Welch ANOVA with Dunnett's T3 multiple comparisons test. (*G*) Unpaired, two-tailed Mann−Whitney test.

In order to fully block transcription of Gm39266 across the +32-kb *Irf8* enhancer, we next carried out two additional approaches. First, we deleted both exons 1 and 2 of Gm39266 to completely eliminate transcription initiation and generated the long promoter deletion (L-pro^–/–^) mice (Fig. 4A–C; Supplemental Fig. S2B). Second, we inserted a 3× polyA signal immediately downstream from exon 2 of Gm39266, right upstream of the +32-kb *Irf8* enhancer, to generate pA/pA mice (Fig. 4D–G; Supplemental Fig. S2C,D). In L-pro^–/–^ mice, Gm39266 transcripts were eliminated, and we found that cDC1s developed normally (Fig. 4B,C). Likewise, in pA/pA mice, the transcription of lncRNA Gm39266 across the +32-kb *Irf8* enhancer was blocked (Fig. 4E). However, development of cDC1 remained undisturbed (Fig. 4F,G). Together, these results indicate that the +41-kb *Irf8* enhancer *cis*-regulates +32-kb *Irf8* enhancer activity independently of lncRNA Gm39266 transcripts and independently of transcription across the +32-kb *Irf8* enhancer region.

### The +41-kb *Irf8* enhancer *cis*-regulates chromatin accessibility and BATF3 binding at the +32-kb *Irf8* enhancer

**Figure 5.**
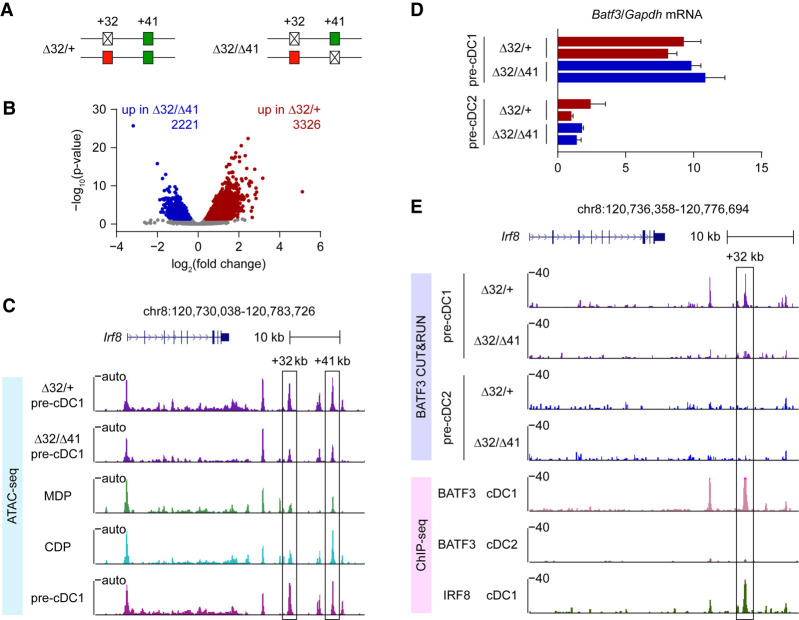
The +41-kb *Irf8* enhancer *cis*-regulates chromatin accessibility and BATF3 binding at the +32-kb *Irf8* enhancer. (*A*) Schematic of Δ32/+ and Δ32/Δ41 mice. (*B*) Volcano plot showing differential ATAC-seq peaks in pre-cDC1s from Δ32/+ and Δ32/Δ41 mice. (*C*) ATAC-seq tracks display the *Irf8* locus in pre-cDC1s from Δ32/+ or Δ32/Δ41 mice, and MDPs, CDPs, or pre-cDC1s from WT mice. The +32- and +41-kb *Irf8* enhancers are shown as boxed. Data shown are one of three similar experiments. (*D*) *Batf3* transcripts, measured by RT-qPCR, in pre-cDC1s and pre-cDC2s from Δ32/+ and Δ32/Δ41 mice. (*E*) CUT&RUN and ChIP-seq tracks display BATF3 and IRF8 binding around the *Irf8* locus in pre-cDC1s or pre-cDC2s from Δ32/+ or Δ32/Δ41 mice, and cDC1s or cDC2s from WT mice. The +32-kb *Irf8* enhancer is shown as boxed. Data shown are one of two similar experiments. Data are mean ± SD.

We previously showed that during cDC1 development, *Irf8* expression first relies on the +41-kb enhancer and later requires the +32-kb enhancer (Bagadia et al. 2019; Durai et al. 2019). Consistently, ATAC-seq analysis demonstrates a dramatic increase in chromatin accessibility at the +32-kb *Irf8* enhancer in pre-cDC1 progenitors compared with MDPs and CDPs (Durai et al. 2019). To explore whether the gain of chromatin accessibility at the +32-kb *Irf8* enhancer is *cis*-regulated by the +41-kb *Irf8* enhancer, we compared the ATAC-seq profile of pre-cDC1 progenitors isolated from Δ32/+ and Δ32/Δ41 mice (Fig. 5A). We chose these genotypes for direct comparison because Δ32/+ and Δ32/Δ41 mice each have only one copy of the +32-kb *Irf8* enhancer, so that the ATAC-seq signal at the +32-kb enhancer region solely reflects its accessibility in the context of a +41-kb enhancer-sufficient (Δ32/+) or -deficient (Δ32/Δ41) allele.

Comparison of the ATAC-seq profiles between pre-cDC1 from Δ32/+ and Δ32/Δ41 mice identified global differences in chromatin accessibility (Fig. 5B). As expected, the +41-kb *Irf8* enhancer region in Δ32/Δ41 mice shows a reduced ATAC-seq signal compared with Δ32/+ mice, consistent with the loss of one copy of the +41-kb *Irf8* enhancer (Fig. 5C). In WT mice, ATAC-seq analysis shows the gain of accessibility at the +32-kb enhancer that occurs in the transition from CDPs to pre-cDC1s. Strikingly, pre-cDC1 progenitors from Δ32/Δ41 mice completely lost this increase in ATAC-seq signal compared with Δ32/+ control mice (Fig. 5C). This result demonstrates that the +41-kb *Irf8* enhancer regulates chromatin accessibility of the +32-kb *Irf8* enhancer region located in *cis*.

Since BATF3 must bind to the +32-kb *Irf8* enhancer to support cDC1 development (Grajales-Reyes et al. 2015), we evaluated the requirement of the +41-kb *Irf8* enhancer in BATF3 binding at the +32-kb enhancer. *Batf3* expression has been shown to be selectively induced in pre-cDC1 but not in pre-cDC2 progenitors (Grajales-Reyes et al. 2015). Importantly, we confirmed that *Batf3* expression in pre-cDC1 progenitors is comparable between Δ32/+ and Δ32/Δ41 mice (Fig. 5D), permitting a direct comparison of the BATF3 binding to the +32-kb *Irf8* enhancer. Using CUT&RUN, we found that BATF3 binding at the +32-kb *Irf8* enhancer occurs only in pre-cDC1 but not in pre-cDC2 progenitors from Δ32/+ mice (Fig. 5E), in agreement with our previous ChIP-seq analysis (Grajales-Reyes et al. 2015). Importantly, we found no BATF3 binding signal by CUT&RUN at the +32-kb *Irf8* enhancer in pre-cDC1 progenitors from Δ32/Δ41 mice (Fig. 5E). This result directly demonstrates that the +41-kb *Irf8* enhancer regulates BATF3 binding to the +32-kb *Irf8* enhancer region in *cis*.

## Discussion

IRF8 is the lineage-determining transcription factor of the cDC1 lineage (Aliberti et al. 2003), which develops through a series of progenitor stages including the MDPs, CDPs, pre-cDC1s, and finally the mature cDC1s (Grajales-Reyes et al. 2015). We found that several *Irf8* enhancers act sequentially to support cDC1 development (Durai et al. 2019; Murakami et al. 2021), starting with the +56-kb enhancer that initiates *Irf8* expression in multipotent progenitor and MDPs. *Irf8* expression increases upon transition to the CDP stage as a result of E-proteins binding to E-box elements in the +41-kb enhancer (Durai et al. 2019). This +41-kb enhancer remains active in mature pDCs, where the E-protein E2-2 maintains high *Irf8* expression (Durai et al. 2019). However, upon transition to the pre-cDC1 progenitor stage, *Irf8* expression becomes dependent on the +32-kb enhancer, where BATF3/Jun heterodimers and IRF8 cooperatively bind to several AP-1-IRF composite elements to support *Irf8* autoactivation (Grajales-Reyes et al. 2015).

We initially suspected that the +41-kb *Irf8* enhancer would act only in pDCs to maintain IRF8 expression based on reporter analysis showing high enhancer activity in pDC, but not in cDC1 or cDC2, subsets (Grajales-Reyes et al. 2015). Surprisingly, however, Δ41/Δ41 mice not only showed impaired IRF8 expression in pDCs but also completely lacked cDC1 development (Durai et al. 2019). In addition, the +41-kb *Irf8* enhancer was required for pre-cDC1 specification, while the +32-kb *Irf8* enhancer was required only for cDC1 maturation. These results indicated that the +41-kb *Irf8* enhancer acts at an earlier stage than the +32-kb enhancer in cDC1 development. Based on the observations that mature cDC1s lost +41-kb enhancer activity in reporter assays (Grajales-Reyes et al. 2015), that mature cDC1s showed reduced chromatin accessibility at the +41-kb enhancer region compared with the CDP stage, and that the +41-kb enhancer cannot maintain normal IRF8 expression in Δ32/Δ32 pre-cDC1 progenitors (Durai et al. 2019), the +41-kb enhancer has been suggested to transiently support *Irf8* expression in the CDP stage, later switching to the +32-kb enhancer in specified pre-cDC1 progenitors and mature cDC1s. Nevertheless, the mechanism for the +41-kb *Irf8* enhancer in cDC1 development has remained unclear.

The +41-kb enhancer could act to increase *Irf8* expression in CDPs for later *Irf8* autoactivation, since the Δ41/Δ41 CDPs show slightly reduced *Irf8* expression compared with WT CDPs (Durai et al. 2019). However, it would be expected to exert an effect in *trans*, since changes in IRF8 protein level would affect both chromosomes. Alternatively, transcription of lncRNA Gm39266 driven by the +41-kb *Irf8* enhancer could induce +32-kb enhancer activity. Finally, a direct enhancer–enhancer interaction could take place in which the +41-kb *Irf8* enhancer directly regulates +32-kb enhance activity independently of lncRNA transcription. These later two alternatives would be expected to exert an effect in *cis*.

In this study, we examined compound heterozygous Δ32/Δ41 mice and found that the activity of the +32-kb *Irf8* enhancer depends on being located in *cis* with a functional +41-kb enhancer. Transcription of the +41-kb enhancer-dependent lncRNA Gm39266 does not mediate subsequent +32-kb enhancer activity. Instead, the +41-kb enhancer modifies accessibility and transcription factor binding to the *cis*-located +32-kb enhancer by a mechanism that does not rely on the transcription of the associated lncRNA. The *cis* interaction between the +41- and +32-kb *Irf8* enhancers raises the possibility that the +41-kb *Irf8* enhancer may remain important throughout cDC1 development after it becomes active. Alternatively, an enhancer switch may occur during cDC1 development, with the +41-kb enhancer driving activation of the +32-kb enhancer and then losing its activity and importance after the +32-kb enhancer becomes active.

Several aspects of the *cis* interaction between *Irf8* enhancers still require future investigations. For instance, whether the +41-kb enhancer regulates the long-distance interactions between the +32-kb enhancer and the *Irf8* promoter or whether the +41-kb enhancer recruits chromatin modifiers to activate +32-kb enhancer activity.

## Materials and methods

### Generation of lncRNA Gm39266 mutant mice

Gm39266 mutant mice were generated as illustrated in Figures 3A and 4, A and D. gRNA 1 (CAGGCACAGTCTGGGTACAC), gRNA 2 (GGTAAGAAATCCTACCTCTG), and gRNA 3 (AGGTTCCATGTCCAGCACAT) were identified using Benchling (https://www.benchling.com/crispr). The ssODN donor sequence used in generating Gm39266 3× polyA knock-in (pA/pA) mice is shown in Supplemental Figure S2D. gRNAs with the desired sequence were ordered from IDT and were conjugated with purified Cas9 protein to form the RNP complex by the Genetic Editing and iPS Cell (GEiC) Center at Washington University in St. Louis. Day 0.5 single-cell zygotes were isolated, and CRISPR reagents were introduced via electroporation by the Department of Pathology/Immunology Transgenic Mouse Core at Washington University in St. Louis. Around 60 single-cell zygotes were electroporated with 8 μM RNP complex using a 1-mm gap cuvette (Bio-Rad). Electroporated zygotes were then transferred into the oviducts of day 0.5 pseudopregnant recipient mice.

The resulting pups were screened by PCR using the primers shown in Supplemental Figure S2, A–C, followed by Sanger sequencing to identify those that had successful deletions or insertions of interest. Mice with the desired mutation were then outcrossed to WT C57BL/6J mice, and the resulting heterozygous mice were intercrossed to generate homozygous Gm39266 mutant mice.

### Mice

Wild-type C57BL/6J mice were obtained from the Jackson Laboratory (000664). *Irf8* +32^−/−^ (Δ32/Δ32) mice (the Jackson Laboratory 032744) (Durai et al. 2019) and *Irf8* +41^−/−^ (Δ41/Δ41) mice (the Jackson Laboratory 032745) (Durai et al. 2019) were generated in-house and described previously.

All mice were maintained on the C57BL/6J background in our specific-pathogen-free facility following institutional guidelines and with protocols approved by the AAALAC-accredited Animal Studies Committee at Washington University in St. Louis. All animals were maintained on 12-h light cycles and housed at 70°F and 50% humidity. Experiments were performed with mice 6–12 wk of age, with sex-matched littermates whenever possible.

### Antibodies and flow cytometry

Flow cytometry and cell sorting were completed on a FACSAria Fusion instrument (BD) and analyzed using FlowJo analysis software (Tree Star). Surface staining was performed at 4°C in the presence of Fc block (2.4G2) in magnetic-activated cell sorting (MACS) buffer (PBS, 0.5% BSA, 2 mM EDTA). Intracellular IRF8 staining was performed using the Foxp3 staining kit (eBioscience 00-5523-00).

The following antibodies were used: CD19 (1D3), CD135 (A2F10.1), MHCII (M5/114.15.2), CD117 (2B8), B220 (RA3-6B2), CD3 (145-2C11), and CD4 (RM4-5) from BD Biosciences; CD3 (145-2C11), CD4 (GK1.5), and MHCII (M5/114.15.2) from Tonbo Biosciences; TER-119 (TER-119), Ly-6G (1A8), B220 (RA3-6B2), CD24 (M1/69), CD115 (AFS98), XCR1 (ZET), CD19 (6D5), CD8α (53-6.7), CD4 (RM4-5), CD11c (N418), and CD3 (17A2) from Biolegend; CD105 (MJ7/18), Siglec-H (eBio440c), CD3 (17A2), CD8α (53-6.7), CD11c (N418), and IRF8 (V3GYWCH) from eBiosciences; and SA-Qdot 605, CD11c (N418), CD317 (eBio927), CD172a (P84), and TCRβ (H57-597) from Invitrogen.

### Isolation of bone marrow progenitors

Bone marrow (BM) progenitors were isolated as described (Bagadia et al. 2019) and depleted of CD3-, CD19-, CD105-, TER-119-, Ly-6G-, and B220-expressing cells by staining with the corresponding biotinylated antibodies, followed by depletion with MagniSort streptavidin-negative selection beads (Thermo Fisher). The remaining lineage^–^ BM cells were then stained with fluorescent antibodies before sorting. CD117^hi^ BM progenitors were identified as lineage^–^CD117^hi^ cells, CDPs were lineage^–^ Siglec-H^−^CD117^int^CD135^+^CD115^+^MHCII^−^CD11c^−^ BM cells, pre-cDC1s were lineage^–^Siglec-H^−^CD117^int^CD135^+^MHCII^int-neg^CD11c^+^CD24^+^ BM cells, and pre-cDC2s were lineage^–^Siglec-H^−^CD117^−^CD135^+^CD115^+^MHCII^−^CD11c^+^ BM cells.

Cells were sorted into Iscove's modified Dulbecco's medium supplemented with 10% FBS, 1% penicillin streptomycin solution, 1% sodium pyruvate, 1% MEM nonessential amino acid, 1% L-glutamine solution, and 55 µM β-mercaptoethanol (complete IMDM).

### Isolation of splenic DCs

Spleens were minced and digested in 5 mL of complete IMDM with 250 µg/mL collagenase B (Roche) and 30 U/mL DNase I (Sigma) for 30 min at 37°C with stirring. After digestion, single-cell suspensions were passed through 70-µm strainers, and red blood cells were lysed with ammonium chloride–potassium bicarbonate (ACK) lysis buffer.

For splenic DC sorting experiments, splenocytes were enriched for CD11c^+^ cells using CD11c microbeads (Miltenyi Biotech). cDC1s were identified as CD317^−^B220^−^MHCII^+^CD11c^+^XCR1^+^CD172a^−^ cells, cDC2s were CD317^−^B220^−^MHCII^+^CD11c^+^XCR1^−^CD172a^+^ cells, and pDCs were CD317^+^B220^+^ cells.

### Retroviral infection and cell culture

Retroviral vector MSCV-Gm39266-IRES-GFP was constructed using the following oligonucleotides: Gm39266_cloneF (ATTAAGATCTACTCTTGAGAGTGAGACTGGACAGT) and Gm39266_cloneR (ATTACTCGAGGATTTAATATAGAACTAGGACATGATAATTACACCCTATAACCTAG). Retroviruses were produced by transfecting retroviral vectors into Plat-E cells as described (Bagadia et al. 2019). CD117^hi^ BM progenitors were sorted, purified, and cultured in complete IMDM supplemented with 5% Flt3L conditioned medium overnight. After removing the culture medium, the cells were transduced with the supernatant containing retroviruses in the presence of 2 µg/mL polybrene by spinoculation at 2000 rpm for 1 h at room temperature. The supernatant containing retroviruses was removed 18 h later, and the infected cells were cultured in complete IMDM supplemented with 5% Flt3L conditioned medium for 7 d before analysis by flow cytometry.

### ATAC-seq and data analysis

ATAC-seq was performed using the Omni-ATAC protocol as previously described (Corces et al. 2017), with modifications. Pre-cDC1 progenitors (5.0 × 10^4^) were sorted from Δ32/+ or Δ32/Δ41 mice; lysed in ice-cold ATAC resuspension buffer (RSB) containing 0.1% NP40, 0.1% Tween-20, and 0.01% digitonin for 3 min at 4°C; and then washed with ATAC-RSB containing only 0.1% Tween-20. Nuclei were spun down by centrifugation and incubated in 50 µL of transposition buffer (25 µL of 2× TD buffer, 16.5 µL of PBS, 0.5 µL of 1% digitonin, 0.5 µL of 10% Tween-20, 5 µL of H_2_O, 2.5 µL of TDE1 Tagment DNA enzyme [Illumina Tagment DNA TDE1 enzyme and buffer kit, Illumina]) for 30 min at 37°C in a thermomixer with 1000 rpm mixing. Transposed DNA was purified with a DNA Clean & Concentrator kit (Zymo Research). ATAC-seq libraries were prepared and data were analyzed as described (Durai et al. 2019).

### CUT&RUN and data analysis

BATF3 CUT&RUN was performed with a CUTANA ChIC/CUT&RUN kit (EpiCypher) per the manufacturer's protocol, with modifications (Liu et al. 2022). To expand the pre-cDC1 and pre-cDC2 progenitors, Δ32/+ and Δ32/Δ41 mice were injected once daily, intraperitoneally, with 15 µg of recombinant Flt-3L-Ig (Bio X Cell BE0098) for eight consecutive days. Pre-cDC1 and pre-cDC2 progenitors were sort purified 24 h after the eighth dose of Flt-3L-Ig treatment. BATF3 CUT&RUN was performed with 1.0 × 10^6^ cells using rabbit anti-BATF3 antibody (Grajales-Reyes et al. 2015). CUT&RUN libraries were prepared and data were analyzed as described (Liu et al. 2022).

### RNA-seq data analysis

RNA-seq data sets were aligned and mapped to the mouse reference genome (GRCm38/mm10) by Bowtie2 software. The duplicated reads were discarded using “make tag directory” of the HOMER software package with the parameter -tbp 1. Data were visualized with “makeUCSCfile” of HOMER (Heinz et al. 2010).

### RT-qPCR

DNase-treated total RNA was prepared with the NucleoSpin RNA XS kit (Macherey-Nagel), and first strand cDNA synthesis was performed with SuperScript IV reverse transcriptase (Invitrogen) using random hexamers (Invitrogen). Relative quantification of gene expression was performed on a StepOnePlus real-time PCR system (Applied Biosystems) using Luminaris Color HiGreen High ROX qPCR master mix (Thermo Scientific). PCR conditions were 2 min at 50°C and 10 min at 95°C, followed by 40 three-step cycles consisting of 15 sec at 95°C, 30 sec at 60°C, and 30 sec at 72°C. Oligonucleotide primers used were as follows: Gm39266_1F (AGGTTTAGGCAAGCTTCACG), Gm39266_2R (ATCTGACAGTGCTGGCTTCA), Gm39266_2F (AGGAAACCCCAGCAACTATG), Gm39266_3R (GCGGATGAATCTGATGCTCT), Gapdh_F (ACGGCCGCATCTTCTTGTGCA), Gapdh_R (ACGGCCAAATCCGTTCACACC), Batf3_F (AGAAGGCTGACAAGCTCCACGA), and Batf3_R (CATCTTCTCGTGCTCCTTCAGC).

### Statistics

Statistical analysis was performed using GraphPad Prism software version 9. Brown−Forsythe and Welch ANOVA with Dunnett's T3 multiple comparisons test and unpaired, two-tailed Mann−Whitney *U*-test were used to determine significant differences between samples.

### Data availability

The ATAC-seq data sets for pre-cDC1 progenitors from Δ32/+ or Δ32/Δ41 mice and BATF3 CUT&RUN data sets for pre-cDC1 or pre-cDC2 progenitors isolated from Δ32/+ or Δ32/Δ41 mice are available in the Gene Expression Omnibus database with accession number GSE218992. RNA-seq data sets for cDC1s, cDC2s, and pDCs used in Figure 2B (GSE127267); ATAC-seq data sets for MDPs, CDPs, and pre-cDC1 progenitors used in Figure 5C (GSE132240); and ChIP-seq data sets for BATF3 or IRF8 in cDC1s or cDC2s used in Figure 5E (GSE66899) can be accessed with the indicated accession numbers.

## Supplementary Material

Supplemental Material
