# Supplementary material for: Cis interactions in the Irf8 locus regulate stage-dependent enhancer activation
Source: Genes Dev. 2023 Apr 1;37(7-8):291–302. doi: 10.1101/gad.350339.122 (PMC10153461; doi:10.1101/gad.350339.122)
Supplement: Supplemental Material [file supp_37_7-8_291__DC1.html]

Cis interactions in the Irf8 locus regulate stage-dependent enhancer activation — Supplemental Material 

# *Cis* interactions in the *Irf8* locus regulate stage-dependent enhancer activation

## Supplemental Material

- Supplemental\_Figures\_S1-S2.pdf
