# Supplementary material for: Cis interactions in the Irf8 locus regulate stage-dependent enhancer activation
Source: Genes Dev. 2023 Apr 1;37(7-8):291–302. doi: 10.1101/gad.350339.122 (PMC10153461; doi:10.1101/gad.350339.122)
Supplement: Supplemental Material [file supp_gad.350339.122_Supplemental_Figures_S1-S2.pdf]

Supplemental Figure S1

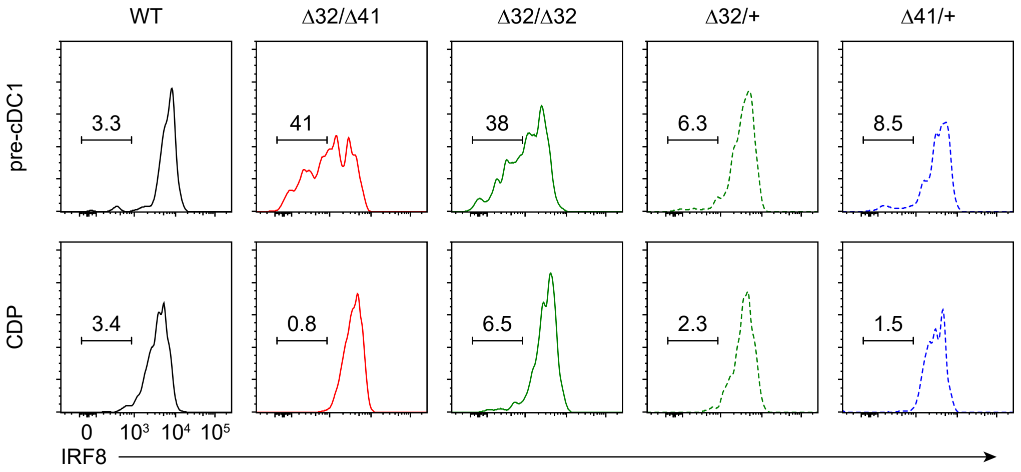

Supplemental Figure S1. Representative flow plots showing intracellular staining for IRF8 in pre-cDC1s (top) and CDPs (bottom) from indicated *Irf8* enhancer mutant mice. Data shown are one of two similar experiments.

Supplemental Figure S2

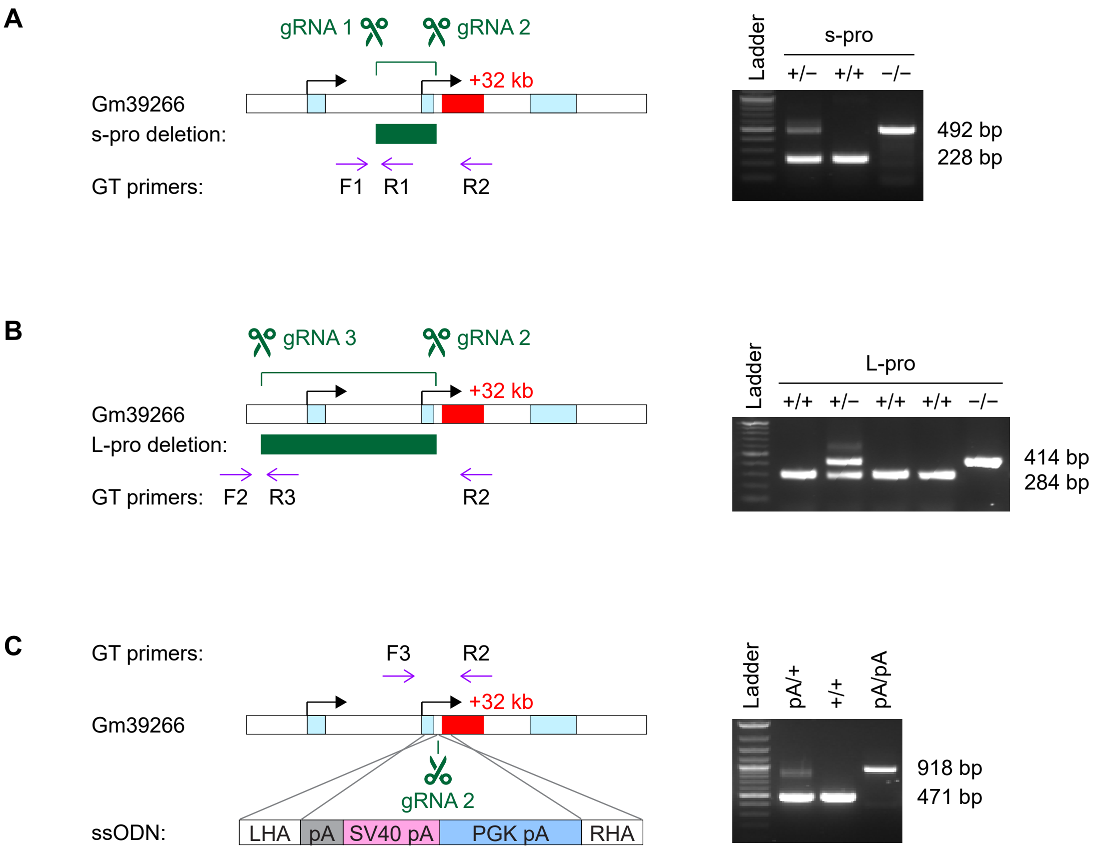

D

```
TATGTTGCAACCTCATGACCCTGCCATGAAGCCAGCACTGTCAGATGCAGGGGGCTGAGGAACACCAGGTCCCAGCAAAG   80
LHA

CATGGTAAGAAATCCTACCTAATAAAAGATCTTTATTTTCATTAGATCTGTGTGTTGGTTTTTTGTGTGGTTTATTGCAG   160
LHA | pA | SV40 pA

CTTATAATGGTTACAAATAAAGCAATAGCATCACAAATTTCACAAATAAAGCATTTTTTTCACTGCATTCTAGTTGTGGT   240
SV40 pA

TTGTCCAAACTCATCAATGTATCTTATCATGTCTAAATTGATGATCTATTAAACAATAAAGATGTCCACTAAAATGGAAG   320
SV40 pA | PGK pA

TTTTTTCCTGTCATACTTTGTTAAGAAGGGTGAGAACAGAGTACCTACATTTTGAATGGAAGGATTGGAGCTACGGGGGT   400
PGK pA

GGGGGTGGGGTGGGATTAGATAAATGCCTGCTCTTTACTGAAGGCTCTTTACTATTGCTTTATGATAATGTTTCATAGTT   480
PGK pA

GGATATCATAATTTAAACAAGCAAAACCAAATTAAGGGCCAGCTCATTCCTCCACTCACGATCTATACTGGGGCCCTGCT   560
PGK pA | RHA

AGGGGTCTCCCCCTTACTTTGCCCCCCTCCCTCACCCAGTAGGTGAGGTGCGATCCTGAAACCAGACCTGCCCCAGGCCA   640
RHA

GTTCCCT                                                                            647
RHA
```

Supplemental Figure S2. Genotyping of lncRNA Gm39266 mutant mice.
(A) Genotyping of Gm39266 exon 2 short promoter deletion (s-pro$^{-/-}$) mice. Green box denotes the 504 bp genomic region (chr8:120,768,912-120,769,415 in GRCm38/mm10) deleted in s-pro$^{-/-}$ mice. The following primers were used for genotyping and illustrated as purple arrows. s-pro_F1: CACTTATGTTGGGTGGCTCA; s-pro_R1: GCAGTGCTCCTCAAATGACA; R2: GGATCCTGCCTGTCAAGGTA.
(B) Genotyping of Gm39266 exon 1-2 long promoter deletion (L-pro$^{-/-}$) mice. Green box denotes the 1656 bp genomic region (chr8:120,768,932-120,770,587 in GRCm38/mm10) deleted in L-pro$^{-/-}$ mice. The following primers were used for genotyping and illustrated as purple arrows. L-pro_F2: GCCTGAACGTCCAGTTCATT; L-pro_R3: CCTGCCTGCAGACTATGACA; R2 as above.
(C) Genotyping of Gm39266 3×polyA knock-in (pA/pA) mice. 3×polyA was inserted 16 bp downstream of Gm39266 exon 2. The following primers were used for genotyping and illustrated as purple arrows. pA_F3: ATTCTGTCTCCCACGTGTCC; R2 as above.
(D) ssODN donor sequence used for generating Gm39266 3×polyA knock-in mice.
